# Rupture of Acute Triple-Barreled Aortic Dissection

**DOI:** 10.7759/cureus.104025

**Published:** 2026-02-21

**Authors:** Atsushi Otani, Hisato Takagi

**Affiliations:** 1 Cardiovascular Surgery, Shizuoka Medical Center, Shimizu, JPN

**Keywords:** aortic aneurysm, aortic dissection, aortic replacement, aortic rupture, false lumen, residual aortic dissection, type-a aortic dissection

## Abstract

We experienced a case of rupture of acute triple-barreled aortic dissection (AD), a rare form of dissection characterized by the presence of two distinct false lumens in addition to the true lumen, involving a dissecting aneurysm of the descending thoracic aorta (DTA). A 71-year-old woman presented with increasing back pain. She had a history of ascending aortic replacement for type-A acute AD, and there was a residual AD from the aortic arch to the bilateral common iliac arteries. An increasing diameter of the DTA had been observed on follow-up CT scans. At the emergency department, her radial pulses were undetectable and the CT scans revealed rupture of the newly developed false lumen of the DTA. Emergency proximal DTA replacement was performed. Intraoperatively, it was confirmed that the cavity of the DTA had a triple-barreled structure. She had wound infection and aspiration pneumonia on postoperative day (POD) 3 and was treated with antibiotics. However, her postoperative course is generally good, and on POD 31, she waits to be discharged to a rehabilitation hospital.

## Introduction

Multibarreled aortic dissection (AD) is known to be a rare type of dissection and is seen in Marfan syndrome. The frequency of multibarreled AD in type-B dissections is reported to be 9%, and it occurs among people without Marfan syndrome [1]. Multibarreled AD often has a poorer prognosis than double-barreled dissection due to its wall fragility. In contrast to the typical double-barreled dissection, which consists of a true lumen and a single false lumen separated by an intimal flap, multibarreled dissection involves two or more distinct false lumens in addition to the true lumen [2]. Furthermore, persistence of a patent false lumen has been identified as an independent risk factor for adverse long-term outcomes, including rupture and dissection-related death [3]. In addition, true lumen compression from multiple false lumens can cause organ malperfusion. Moreover, there are no clear standards for its treatment strategy. We experienced a survived case of rupture of acute triple-barreled AD on dissecting descending thoracic aortic aneurysm, and the characteristics and treatment methods of this disease were discussed.

## Case presentation

**Figure 1:**
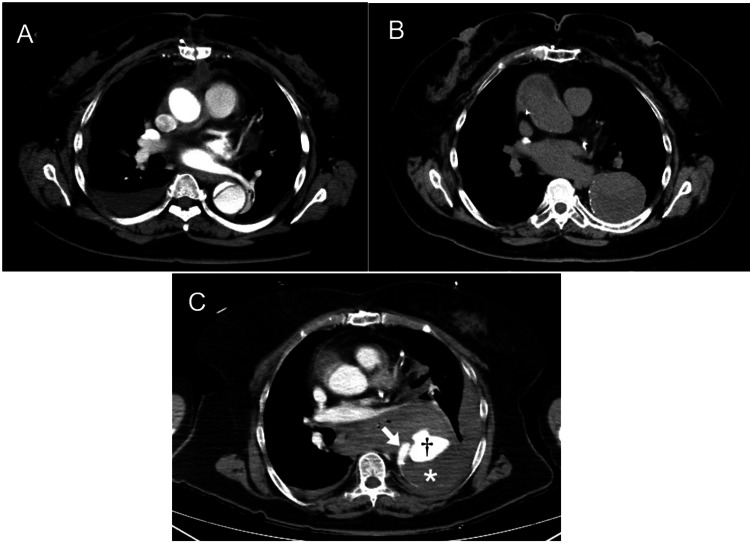
A: CT scan showed a residual dissection after the initial operation. B: Follow-up CT scan showed an increasing diameter of the descending thoracic aorta. C: Contrast-enhanced CT scan revealed rupture (†) of the newly formed false lumen in the triple-barreled dissection. The arrow indicates the true lumen; the asterisk (*) indicates the previous false lumen; and the dagger (†) indicates the newly formed false lumen.

A 71-year-old woman presented with increasing back pain. She had no physical characteristics of Marfan syndrome or other connective tissue diseases. She had a history of ascending aortic replacement for type-A acute AD 12 years before. There was a residual AD from the aortic arch to the bilateral common iliac arteries (Figure 1A). An increasing diameter of the descending thoracic aorta (DTA) had been observed on follow-up CT scans (Figure 1B). On arrival, SpO₂ was 99% with a 10 L non-rebreather oxygen mask, and the heart rate was 86 bpm. Bilateral radial pulses were absent, and blood pressure could not be measured, consistent with hemodynamic shock. The Glasgow Coma Scale score was 13 (E4V4M5). No focal neurological deficits or limb ischemia were observed. Blood examination revealed mild anemia, mildly elevated white blood cell count and C-reactive protein levels, elevated D-dimer levels, normal activated partial thromboplastin time, and slightly prolonged prothrombin time-international normalized ratio (Table 1).

**Table 1:** Laboratory findings on admission. APTT: Activated partial thromboplastin time

Parameter	Result	Unit	Reference range
Hemoglobin	9.3	g/dL	13.7–16.8
White blood cell count	10,800	/µL	3,300–8,600
C-reactive protein	5.4	mg/dL	<0.14
D-dimer	24.2	µg/mL	<1.0
APTT	27	sec	25–35
PT-INR	1.21	–	0.80–1.20

Contrast-enhanced CT scans revealed rupture of the newly developed false lumen of the DTA (Figure 1C). The maximum diameter of the DTA was 66 mm. Emergency proximal DTA replacement was executed. The time from symptom onset to surgery was approximately six hours.

**Figure 2:**
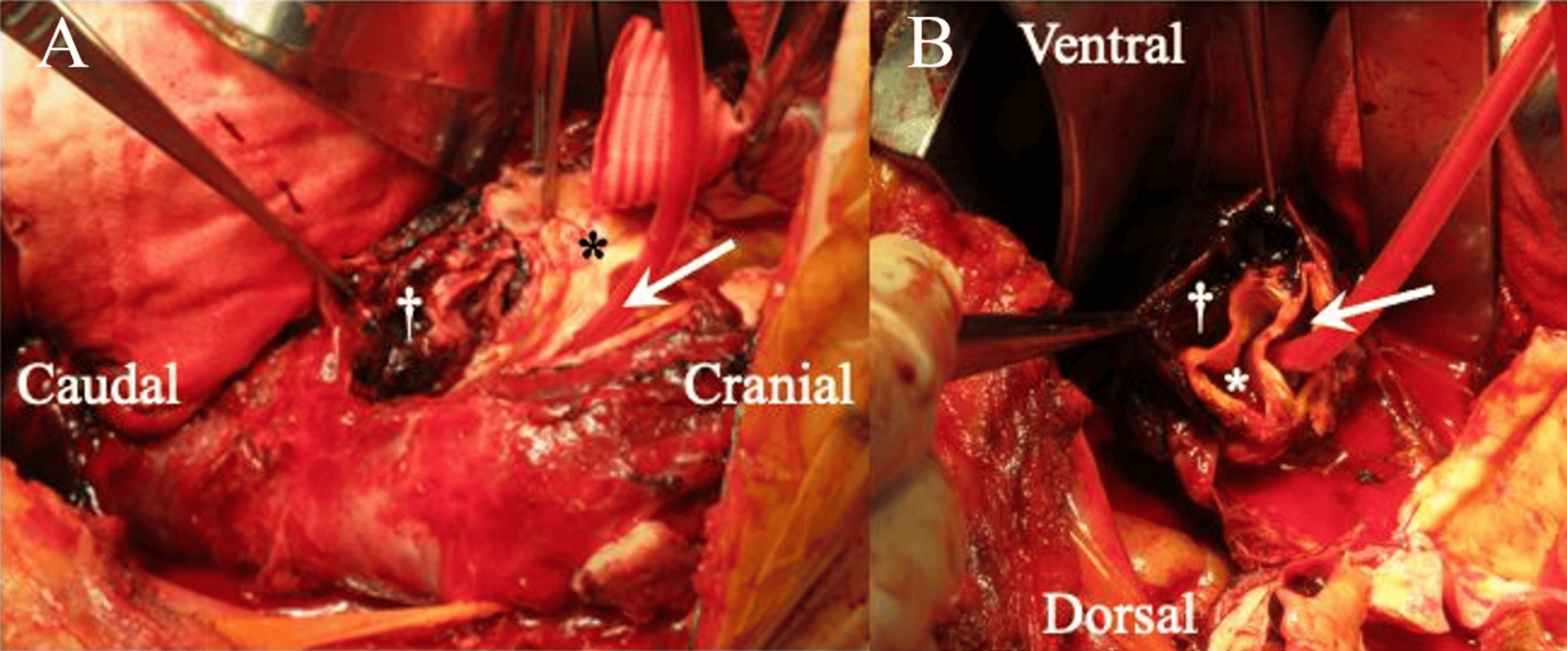
Intraoperative findings. (A) Longitudinal view of the descending thoracic aorta demonstrating the triple-barreled configuration. (B) Cross-sectional view showing the true lumen and two distinct false lumens. The arrow indicates the true lumen; the asterisk (*) indicates the previous false lumen; and the dagger (†) indicates the newly formed false lumen.

The surgery in the right lateral decubitus position was performed under general anesthesia. The thoracotomy was done at the 4th intercostal space, and the DTA with hematoma was exposed. Total cardiopulmonary bypass via the femoral artery and vein was established. The DTA was opened distally to the origin of the left subclavian artery under moderate hypothermic circulatory arrest. The aorta had a triple-barreled structure (Figure 2). A 26 mm straight graft (TRIPLEX) with one branch was anastomosed to the true lumen of the transected aorta just distally to the origin of the left subclavian artery, placing felt strips on the outer and inner sides. The distal anastomosis was performed in the same fashion at the level of Th9.

On postoperative day (POD)3, wound infection in the right inguinal region and aspiration pneumonia were diagnosed and treated using vacuum-assisted closure and antibiotics, respectively. However, the postoperative course is generally good, and the patient is now waiting to be discharged to a rehabilitation hospital on POD 31.

## Discussion

Although Marfan syndrome was previously reported to be a risk factor for triple-barreled AD [4], no clear correlation has been reported in a recent study [1]. The present patient did not have Marfan syndrome. There are no obvious differences in background characteristics between double- and triple-barreled dissections [1]. The second newly formed false lumen is often caused by a tear in the media of the first false lumen. Therefore, the second false lumen has a thinner wall than the first false lumen, which makes triple-barreled dissection more fragile than double-barreled dissection. The present case seemed to be caused by the same mechanism based on CT scans, surgical, and pathological findings. In addition, the two false lumens may compress the true lumen from both sides, causing organ malperfusion, and the prognosis is poorer than in double-barreled dissection. Norton et al. [5] reported that the median time from initial dissection to diagnosis of triple-barreled dissection was 4.2 years. At the time of diagnosis of triple-barreled dissection, 85% of patients required urgent aortic repair for rapid growth (36%), aortic diameter ≥55 mm (30%), malperfusion (6%), intractable pain (6%), and rupture/type A (6%). Thirty-day mortality after triple-barreled dissection was 12%. In the present case, the triple-barreled dissection with rupture was diagnosed, resulting in emergency surgery 12 years after the initial dissection.

Specific treatment criteria for triple-barreled dissection have not been determined. Emergency surgery was performed because the DTA had already ruptured, and a triple-barreled dissection was confirmed during the surgery in the present case. What could have been done to prevent the aortic rupture? Postoperative contrast-enhanced CT after the initial dissection revealed residual dissection extending to both common iliac arteries. Aortic events are more in acute aortic dissection (AAD) patients with a patent residual false lumen than in those with a completely thrombosed false lumen (hazard ratio 5.43, p<0.001) [6]. In this patient, progressive enlargement of the DTA on follow-up CT, together with the development of a newly formed false lumen and markedly elevated inflammatory markers, may have reflected increasing wall stress and instability, ultimately culminating in rupture. Elevated admission CRP has been associated with increased short- and mid-term mortality in AAD, further supporting the role of inflammatory activation in adverse aortic remodeling [7]. It is important not to leave the false lumen patent. Total arch replacement instead of ascending replacement for the initial type A dissection may have promoted thrombosis in the false lumen and prevented the aortic enlargement. In addition, as shown in the Instead-XL trial [8], thoracic endovascular aortic repair is effective for subacute and chronic type B dissection. Prophylactic endovascular repair after the initial surgical repair may have prevented rupture of the aorta in the present case.

## Conclusions

In this case, long-term persistence of a residual false lumen was associated with progressive aortic enlargement and eventual rupture 12 years after the initial dissection. This experience suggests that triple-barreled AD may be associated with a higher risk of adverse outcomes than double-barreled AD, underscoring the importance of careful long-term surveillance and consideration of proactive interventions, including strategic initial surgery and endovascular repair when appropriate.
